# Integrative analysis of circRNA, miRNA, and mRNA profiles to reveal ceRNA regulation in chicken muscle development from the embryonic to post-hatching periods

**DOI:** 10.1186/s12864-022-08525-5

**Published:** 2022-05-03

**Authors:** Qiuxia Lei, Xin Hu, Haixia Han, Jie Wang, Wei Liu, Yan Zhou, Dingguo Cao, Fuwei Li, Jie Liu

**Affiliations:** 1 Poultry Institute, Shandong Academy of Agricultural Sciences, Ji’nan, 250023 China; 2 Poultry Breeding Engineering Technology Center of Shandong Province, Ji’nan, 250023 China; 3 Institute of Animal Sciences, Chinese Academy of Agricultural Sciences, Beijing, 100193 China; 4 Molecular and Cellular Biology, Gembloux Agro-Bio Tech, University of Liège, 5030 Gembloux, Belgium

**Keywords:** Circular RNA, ceRNA, Chicken, Breast muscle, Development

## Abstract

**Background:**

The growth and development of skeletal muscle are regulated by protein-coding genes and non-coding RNA. Circular RNA (circRNA) is a type of non-coding RNA involved in a variety of biological processes, especially in post-transcriptional regulation. To better understand the regulatory mechanism of circRNAs during the development of muscle in chicken, we performed RNA-seq with linear RNA depletion for chicken breast muscle in 12 (E 12) and17 (E 17) day embryos, and 1 (D 1), 14 (D 14), 56 (D 56), and 98 (D 98) days post-hatch.

**Results:**

We identified 5755 differentially expressed (DE)-circRNAs during muscle development. We profiled the expression of DE-circRNAs and mRNAs (identified in our previous study) at up to six time points during chicken muscle development and uncovered a significant profile (profile 16) for circRNA upregulation during aging in muscle tissues. To investigate competing endogenous RNA (ceRNA) regulation in muscle and identify muscle-related circRNAs, we constructed a circRNA-miRNA-mRNA regulatory network using the circRNAs and mRNAs from profile 16 and miRNAs identified in our previous study, which included 361 miRNAs, 68 circRNAs, 599 mRNAs, and 31,063 interacting pairs. Functional annotation showed that upregulated circRNAs might contribute to glycolysis/gluconeogenesis, biosynthesis of amino acids, pyruvate metabolism, carbon metabolism, glycogen and sucrose metabolism through the ceRNA network, and thus affected postnatal muscle development by regulating muscle protein deposition. Of them, circRNA225 and circRNA226 from the same host gene might be key circRNAs that could regulate muscle development by interacting with seven common miRNAs and 207 mRNAs. Our experiments also demonstrated that there were interactions among circRNA225, gga-miR-1306-5p, and heat shock protein alpha 8 (HSPA8).

**Conclusions:**

Our results suggest that adequate supply of nutrients such as energy and protein after hatching may be a key factor in ensuring chicken yield, and provide several candidate circRNAs for future studies concerning ceRNA regulation during chicken muscle development.

**Supplementary Information:**

The online version contains supplementary material available at 10.1186/s12864-022-08525-5.

## Background

The broiler industry is a key player in contributing to sustainable food sources worldwide. Skeletal muscle is the most important component of the bird and directly correlates with meat production and quality in the broiler industry. Therefore, unveiling the molecular mechanisms underneath skeletal muscle formation and development is of vital interest. Muscle development is a complex process that can be regulated by several genes, transcription factors, and some non-coding RNAs [1–5].

CircRNAs are a novel type of non-coding RNA that comprise a class of RNA covalently closed-loop structures without 5′–3′ polarities. They are created by RNA splicing events that occur at a characteristic “head to tail” splice junction, where an acceptor splice site at the 5′ end of an exon and a donor site at the 3′ end of a downstream exon are joined [6]. CircRNAs can regulate gene expression at a transcriptional, post-transcriptional, or translational level by the use of microRNA sponges [7]. Increasing evidence has demonstrated that circRNAs modulate gene expression during processes of myogenesis in chicken. CircSVIL promotes both myoblast proliferation and differentiation by sponging miR-203 and increasing the expression of its targets, Jun proto-oncogene (c-JUN) and myocyte enhancer factor 2C (MEF2C) [7]. CircFGFR2 promotes proliferation and differentiation in chicken skeletal muscles by acting as a decoy for miR-133a-5p and miR-29b-1-5p [8]. However, the completed catalog of circRNAs involved in the muscle development of chickens remains mostly unknown. Ouyang et al. (2017) identified 462 differentially expressed (DE) circRNAs during embryonic leg muscle development in chicken [9].

Muscle growth and development go through two important periods: hyperplasia and hypertrophy. Hyperplasia refers to increases in the number of cells or muscle fibers that occur mainly in the embryonic period. Whereas, hypertrophy refers to increases in cell size that occur mainly after birth [10]. Our previous study on a protein-coding gene expression profile in the breast muscle of Shouguang chickens of 12 (E 12) and 17 (E 17) day embryos and at 1 (D 1), 14 (D 14), 56 (D 56), and 98 (D 98) days post-hatch also showed that there were distinct molecular processes that occurred in chicken muscle development between embryonic and post-hatching periods [11]. The systematic identification and characterization of circRNAs from the embryonic to post-hatching periods will contribute to a completed catalog of circRNAs in chicken muscle development and in uncovering the regulatory mechanisms of muscle development.

## Methods

### Animals and sample collection

One thousand Shouguang chicken eggs were obtained from an experimental farm of the Poultry Institute, Shandong Academy of Agricultural Sciences (PI, SAAS, Ji’nan, China). All eggs were incubated by a normal procedure and chicks were reared in cages under continuous lighting using standard conditions of temperature, humidity, and ventilation on the PI, SAAS, farm. The same diet was fed to all chickens and a three-phase feeding system was used: the starter ration (d 1 to d 28) with 21.0% crude protein and 12.12 MJ/kg, the second phase (d 28 to d 56) with 19.0% crude protein and 12.54 MJ/kg, and the last phase (after d 56) with 16.0% crude protein and 12.96 MJ/kg. Feed and water were provided ad libitum during the experiment. Breast muscles were used at E 12, E 17, D 1, D 14, D 56, and D 98. The embryos, bodies and breast muscles on both sides were weighed. The data were analyzed by one-way ANOVA and multiple comparison using SAS 9.4, and the results were shown as means ± SD. Sub-samples were immediately fixed in 4% paraformaldehyde and held at room temperature and additional samples were snap frozen in liquid nitrogen and held at -80 °C until RNA extraction [11].The sex of the chickens was determined by PCR amplification using sex-specific primers. Chickens with two bands of 450 and 600 bp were females, whereas those with one band of 600 bp were males [12]. Eighteen female chickens from the six developmental stages (E12, E17, D1, D14, D56, and D98) were used for sequencing. Three biological replicates for each stage.

### Histology

Fixed tissues were dehydrated through an ascending ethanol series, embedded in paraffin, and sectioned (3–5 μm). After dewaxing in xylene and rehydration using a descending alcohol gradient, mounted muscle sections were stained with hematoxylin and eosin (H&E). Images were captured and processed with Image-Pro Plus 6.0 software [13].

### CircRNA library construction and Illumina sequencing

Total RNA was isolated and purified using TRIzol reagent (Invitrogen, Carlsbad, CA, USA) following the manufacturer's procedure. The amount and purity of the RNA for each sample were quantified using a NanoDrop ND-1000 (NanoDrop, Wilmington, DE, USA). The RNA integrity was assessed by an Agilent 2100 with RIN number > 7.0 [11]. Approximately 5 μg of total RNA was used to deplete ribosomal RNA according to the protocol of a Ribo-Zero rRNA Removal Kit (Illumina, San Diego, USA). Then, RNAs were treated with RNase R (Epicenter Inc, Madison, WI, USA) to remove linear RNAs and to enrich circRNAs. After removing ribosomal and linear RNAs, the enriched circRNAs were fragmented into small pieces using divalent cations under a high temperature. Then the cleaved RNA fragments were reverse-transcribed to create the cDNA, which was then used to synthesize U-labeled second-stranded DNAs with *E. coli* DNA polymerase I, RNase H, and dUTP. An A-base was then added to the blunt ends of each strand to prepare for the ligation to indexed adapters. Each adapter contained a T-base overhang for ligating the adapter to the A-tailed fragmented DNA. Single- or dual-index adapters were ligated to the fragments, and size selection was performed with AMPureXP beads. After the heat-labile UDG enzyme treatment of the U-labeled second-stranded DNAs, the ligated products were amplified by PCR using the following conditions: initial denaturation at 95 °C for 3 min; eight cycles of denaturation at 98 °C for 15 s, annealing at 60 °C for 15 s, and extension at 72 °C for 30 s; and then a final extension at 72 °C for 5 min. The average insert size for the final cDNA library was 300 ± 50 bp. Finally, we performed paired-end sequencing on an Illumina Hiseq 4000 (LC Bio, Hangzhou, China) following the vendor's recommended protocol.

### RNA-seq data analysis

Cutadapt [14] was used to remove the reads that contained adaptor contamination, low quality, and undetermined bases. Then, sequence quality was verified using FastQC (http://www.bioinformatics.babraham.ac.uk/projects/fastqc/). We used Bowtie2 [15] and Tophat2 [16] to map reads to the chicken reference genome (Gallus-gallus-5.0/galGal5). The remaining reads (unmapped reads) were mapped to the genome using TopHat-Fusion [17]. CIRCExplorer [18, 19] was used to de novo assemble the mapped reads to circRNAs. Then, back-splicing reads were identified in unmapped reads by TopHat-Fusion and CIRCExplorer. All samples generated unique circRNAs. The DE circRNAs were selected with log_2_(fold change) > 1 or log2 (fold change) < -1 and with statistical significance (*p*-value < 0.05) by R package-edge R [20].

### Time series expression profile clustering

Co-expression circRNAs and mRNAs were clustered by Short Time-Series Expression Miner (STEM, version 1.3.11) [21]. Expression profiles of circRNAs and mRNAs were clustered based on their log_2_(normalized data) and their correlation coefficients. The maximum unit change in model profiles between time points was adjusted to two and the maximum number of model profiles to 20. The statistical significance of the number of circRNAs and mRNAs to each profile versus the expected number was computed using the algorithm proposed by Ernst and Bar-Joseph [21].

### Construction of the CircRNA-miRNA-mRNA Network

MicroRNA target sites in exons of circRNA loci and target mRNAs of miRNAs were identified using TargetScan and miRanda (http://www.microrna.org/microrna/home.do) with a score of 50 or higher and free energy -10 or lower [22]. The circRNA-miRNA-mRNA network was constructed according to the prediction of miRNA binding sites. Cytoscape software was used to construct circRNA-miRNA-mRNA networks [23]. GO annotation and KEGG pathway [24–26] analysis were implemented in Omicstudio (https://www.omicstudio.cn/index) for mRNAs regulated by circRNAs through ceRNA.

### Cell culture

293T (human embryonic kidney) cells obtained from ATCC (Cell Systems & cGMP Biorepository, Gaithersburg, MD, USA) and were cultured in DMEM (Gibco, Gaithersburg, MD, USA) supplemented with 10% fetal bovine serum (Hyclone, Logan, UT, USA), 1% penicillin/streptomycin (Invitrogen, Carlsbad, CA, USA). All cells were cultured at 37 °C in a 5% CO2 humidified atmosphere.

### Dual-luciferase reporter assay

For luciferase reporter assay, 293T cells were seeded in 48-well plates and co-transfected with wild type (WT) or mutated (MT) reporter vector and miR-1306-5p mimics or NC duplexes. Firefly and Renilla luciferase activities were measured at 48 h post transfection using a Dual-GLO Luciferase Assay System Kit (Promega, Madison, USA), following the manufacturer’s instructions. Luminescence was measured using a Fluorescence/Multi-Detection Microplate Reader (BioTek, Vermont, USA) and firefly luciferase activities were normalized to Renilla luminescence in each well.

### Quantitative real-time PCR analysis

The circRNAs were validated with convergent and divergent primers according to a previous study [27]. Details of divergent and convergent primers are in Table S1. PCR products of divergent and convergent primers for cDNA and genomic DNA were analyzed by agarose gel electrophoresis. Back-splicing sites of circRNAs were confirmed by Sanger sequencing at Tsingke Biotech Co. Ltd. (Beijing, China). To check the sensitivity of circRNA to RNaseR, qRT-PCR was also performed using RNA samples with and without RNaseR treatment. The expression of PCR products from divergent primers for each sample was validated using qRT-PCR. The qRT-PCR program was implemented using ABI7500 (Life Technologies, Carlsbad, USA) with SYBR Green (TaKaRa, Dalian, China) in a final volume of 20 μL. Each assay was performed in triplicate using the following cycling conditions: 95 °C/30 s and 40 cycles of 95 °C/5 s, and 60 °C/34 s. The ^△△^Ct method was used to compare gene expression, with* β* actin as a reference gene. Three independent replications were used for each assay and data were presented as means ± SD.

## Results

### Characteristics of the body weight and breast muscle dynamic variation

**Fig. 1:**
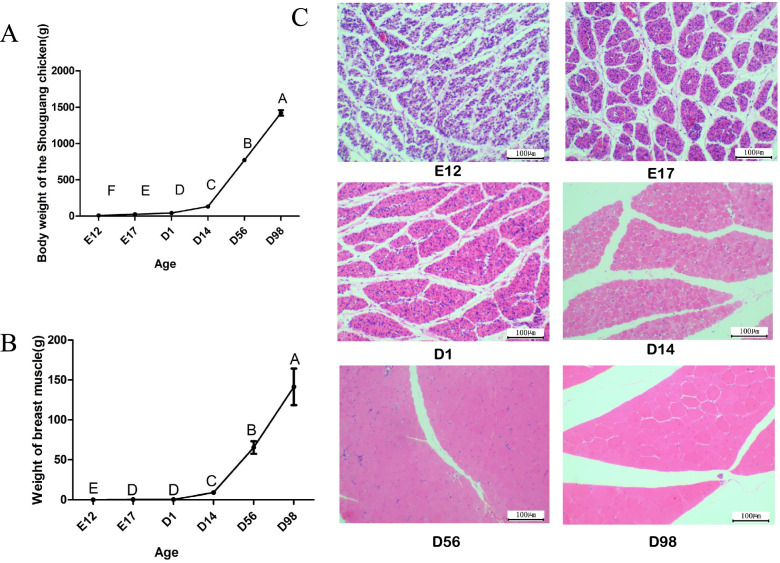
Changes in body weights, muscle weights and fibers at different ages of Shouguang chickens. **A** Changes in body weight with age. **B** Changes in breast muscle with age. **C** Muscle fibers of the breast muscle stained with hematoxylin and eosin (H&E) (bar, 100 μm). Different uppercase superscripts in (**A**) and (**B**) indicate significant differences in body weight and breast muscle weight at different ages (*P* < 0.01)

To investigate the muscle development of chicken, we explored the body weight and breast muscle dynamic variation of Shouguang chickens from the embryonic to post-hatching periods (E12, E17, D1, D14, D56, and D98). The result showed that the body weight (BW) and breast muscle weight (BrW) was increased with growth of chicken, and the BW and BrW were increased markedly after hatching, especially from D14 to D98 (Fig. 1A-B). Moreover, the muscle tissues of chickens were performed using hematoxylin–eosin (H-E) staining to visual the muscle fibers from the embryonic to post-hatching periods (Fig. 1C).

### Overview of CircRNA deep sequencing data

**Fig. 2:**
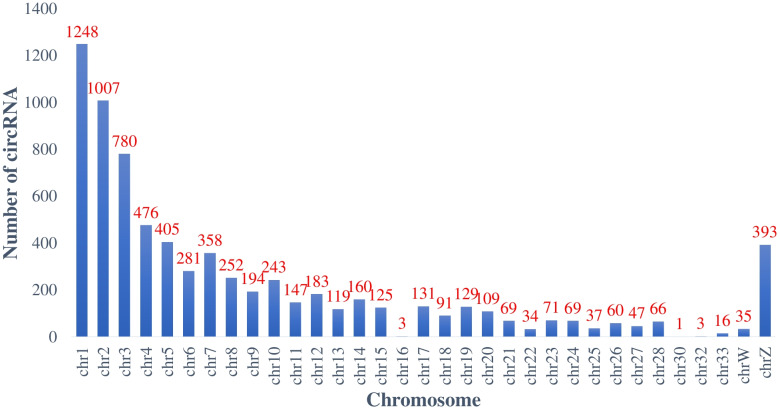
Distribution of circRNAs on chicken chromosomes

To identify the circRNA profile during skeletal muscle development in chickens, breast muscle tissues of three female Shouguang chickens at E 12, E 17, D 1, D 14, D 56, and D 98 were used for RNA sequencing after rRNA-depletion and RNase R treatment. For RNA-seq, a total of 1,310,663,702 reads were generated from 18 samples, and each sample yielded more than 60 million reads. The average GC content was 55.72% (Table 1). Raw data were processed to remove adapter and low quality sequences and then mapped to the chicken reference genome (Gallus-gallus-5.0/galGal5). From the 18 muscle tissues, a total of 30,840 circRNAs were detected. To generate a confident set of circRNAs, we only retained circRNAs expressed in at least three or more samples. These steps resulted in 7,342 circRNAs, and all the circRNAs originated from 2,291 chicken genes (Table S2). These circRNAs were located on chicken chromosomes 1–28, 30, 32, 33, W, and Z (Fig. 2). We identified 5,755 circRNAs that were DE in pairwise comparisons between the libraries of breast muscle at the six developmental stages (*p* < 0.05, log_2_(fold change) ≥ 1 or log_2_(fold change) ≤ -1) (Table S3).

**Table 1:** Summary of sequencing results in chicken breast muscle at six stages

Sample	Raw Read	Valid Read	Valid Ratio (%)	Q20 (%)	Q30 (%)	GC content (%)
E12_1	79,361,516	78,494,064	98.91	99.59	95.12	61.50
E12_2	70,330,506	69,323,190	98.57	99.33	94.31	61.00
E12_3	71,844,158	70,861,754	98.63	99.49	94.80	59.50
E17_1	91,378,212	90,116,164	98.62	99.55	94.98	63.50
E17_2	76,521,826	75,503,870	98.67	99.42	94.47	63.00
E17_3	74,165,582	72,949,882	98.36	99.56	94.99	62.50
D1_1	75,095,908	74,183,634	98.79	99.26	94.35	61.00
D1_2	73,852,734	72,432,066	98.08	99.55	94.91	60.50
D1_3	80,960,850	79,890,708	98.68	99.55	95.03	61.50
D14_1	67,738,396	67,005,658	98.92	99.67	95.74	51.00
D14_2	67,411,076	65,705,194	97.47	99.59	96.61	50.50
D14_3	65,436,016	63,721,900	97.38	99.60	96.31	50.00
D56_1	67,920,060	66,227,126	97.51	99.56	96.46	49.00
D56_2	73,784,802	72,771,396	98.63	99.63	95.70	47.50
D56_3	67,654,772	66,716,740	98.61	99.62	95.16	50.00
D98_1	68,319,518	65,730,624	96.21	99.55	96.62	49.00
D98_2	71,561,460	70,704,572	98.80	99.74	95.58	51.00
D98_3	67,326,310	64,822,898	96.28	120.72	116.80	51.00
Total	1,310,663,702	1,287,161,440				55.72%

### STEM analysis of DE-circRNAs and DE-mRNAs

**Fig. 3:**
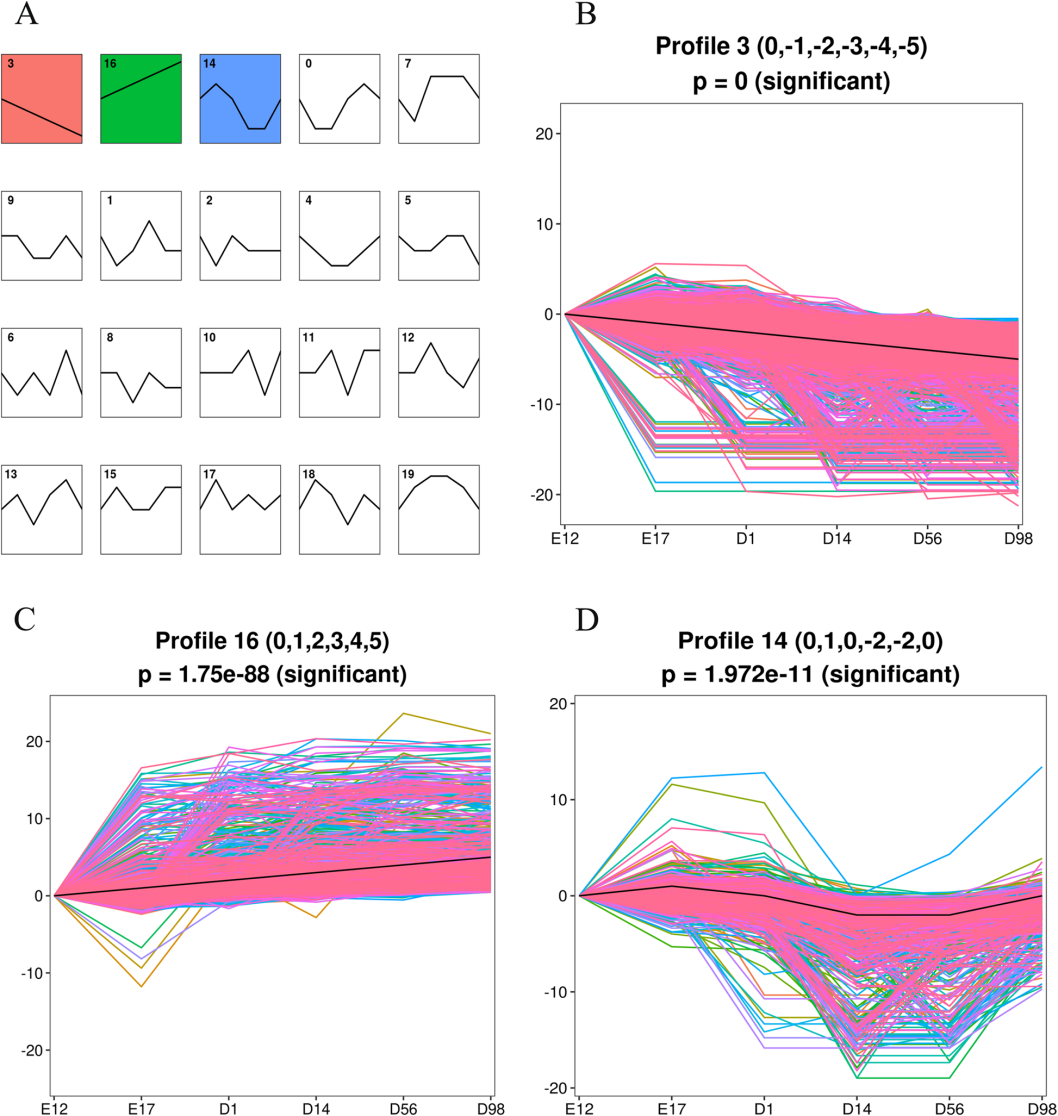
STEM analysis of transcript profiles. **A** Each box corresponds to a type expression profile and only colored profiles are significantly different. **B** Profile 3. **C** Profile 16. **D** Profile 14

We investigated the DE-mRNAs in breast muscle at E12, E17, D1, D14, D56, and D98 in a previous study [11], and identified 11,975 DE-mRNAs (Table S4). CircRNA can regulate mRNA expression. There may be a regulatory relationship between circRNAs and mRNAs with the same expression pattern. Therefore, we performed trend analysis using STEM to identify DE-circRNAs and DE-mRNAs with the same expression pattern during breast muscle development. We identified three significant profiles (Fig. 3A). Profile 3 with a downregulated pattern contained one circRNA and 5,018 mRNAs (Fig. 3B and Table S5). Profile 16 with an upregulated pattern contained 69 circRNAs and 1,173 mRNAs (Fig. 3C and Table S6). Profile 14, with two circRNAs and 722 mRNAs was the third pattern. There was an increase in regulation from E12 to E17 and a decrease from E17 to D14, and it remained stable from D14 to D56 and finally increased from D56 to D98 (Fig. 3D and Table S7).

### Exploration of the CircRNA-mediated CeRNA regulatory network

Increasing evidence indicates that circRNAs can sequester relevant miRNAs through MREs to post-transcriptionally regulate gene expression. We identified 587 miRNAs in breast muscle at E12, E17, D1, D14, D56, and D98 in a previous study [28] (Table S8). To investigate ceRNA regulation in muscle and identify muscle-related circRNAs, we identified putative miRNA-circRNA and miRNA-mRNA interactions using the 587 miRNAs and transcripts identified in profiles 3, 14, and 16. Due to the small number of circRNAs in profiles 3 (one circRNA) and 14 (two circRNAs), a ceRNA network was not constructed with these profiles. In profile16, we obtained 823 miRNA-circRNA and 15,474 miRNA-mRNA interaction pairs (Table S9) and constructed a circRNA-miRNA-mRNA triple network, which included 361 miRNAs, 68 circRNAs, 599 mRNAs, and 31,063 interaction pairs (Table S10). Interestingly, in profile 16 we observed that two circRNA isoforms (circRNA225, circRNA226) had the same host gene, glycerol-3-phosphate dehydrogenase 2 (GPD2). The regulatory network showed that circRNA225 and circRNA226 co-regulated seven miRNAs (gga-miR-106-3p, gga-miR-12239-5p, gga-miR-12283-3p, gga-miR-1306-5p, gga-miR-1773-3p, gga-miR-183, and gga-miR-3594-3p) and 207 mRNAs through ceRNA regulation (Table S11).

### Functional analysis of the CircRNAs during muscle development

**Fig. 4:**
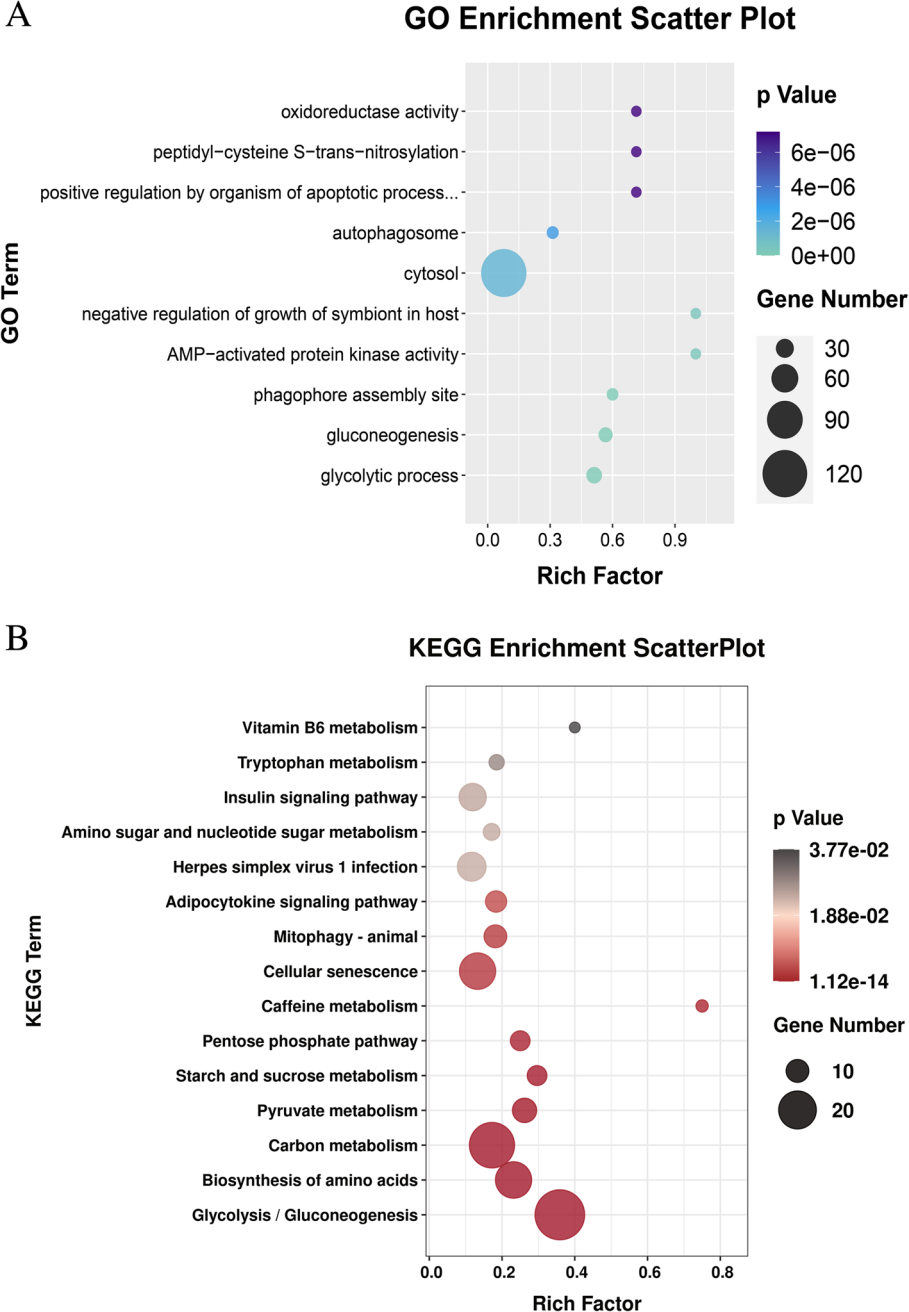
Function annotation of the circRNAs in profile 16. **A** Top 10 GO terms of the putative target mRNAs in the ceRNA network using transcripts in profile 16. **B** Top 15 pathways of the putative target mRNAs in the ceRNA network using transcripts in profile 16

To investigate the potential functional implication of the circRNAs during breast muscle development, we performed GO and KEGG analysis of the mRNAs in the circRNA-miRNA-mRNA triple network constructed with profile 16 (Table S12). GO functional annotation showed that upregulated circRNAs in profile 16 contributed to the glycolytic process, gluconeogenesis, AMP-activated protein kinase activity, peptidyl-cysteine S-trans-nitrosylation, and oxidoreductase activity (Fig. 4A). The KEGG pathway enrichment displayed that glycolysis/gluconeogenesis, biosynthesis of amino acids, pyruvate metabolism, carbon metabolism, glycogen and sucrose metabolism were the top pathways (Fig. 4B). These results showed that the expression of the circRNAs that regulated metabolism increased with increased age, and they may play an important role in muscle development.

### Validation of CircRNAs

**Fig. 5:**
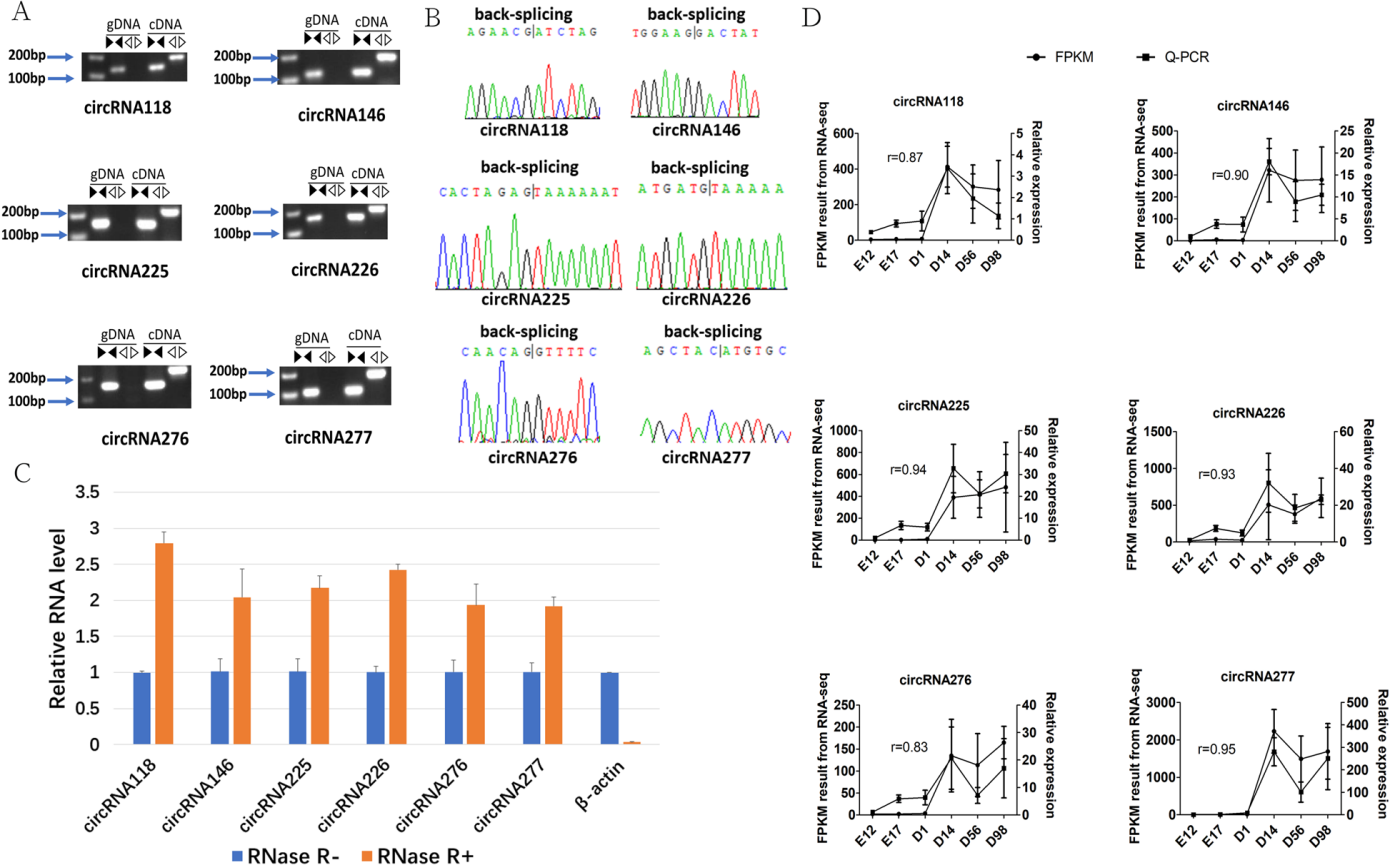
Experimental validation of circular RNAs. **A** Divergent primers amplify circRNAs in cDNA, but not genomic DNA (gDNA). Black triangles represent convergent primers and white triangles represent divergent primers. The gels were cropped to improve the clarity and conciseness of the presentation (**B**) Sanger sequencing confirmed the back-splicing junction sequence of circRNAs. **C** qRT-PCR showing resistance of circRNAs to RNaseR digestion. (**D**) The qRT-PCR validation of six differentially expressed circRNAs in six development time points

Six circRNAs were randomly selected and the back-site junctions were verified by agarose gel electrophoresis, Sanger sequencing, and quantitative real-time PCR (qRT-PCR) (Fig. 5). We designed divergent and convergent primers for each circRNAs, and used both genomic DNA (gDNA) and complementary DNA (cDNA) as templates for PCR. Divergent primers from each circRNA amplified the expected fragments using cDNA as a template, but they could not amplify PCR products using the gDNA template, suggesting the presence of back-site junctions (Fig. 5A), which were also validated by Sanger sequencing (Fig. 5B). We quantified the six candidate circRNAs with RNaseR treatment to detect the resistance of circRNA to the digestion by RNaseR. The result showed that circRNAs had much more resistant than the linear mRNA (Fig. 5C). The qRT-PCR validation for the five circRNAs was consistent with the trends obtained from circRNA sequencing data (Fig. 5D).

### Verification of the interaction among circRNA, miRNA, and mRNA

**Fig. 6:**
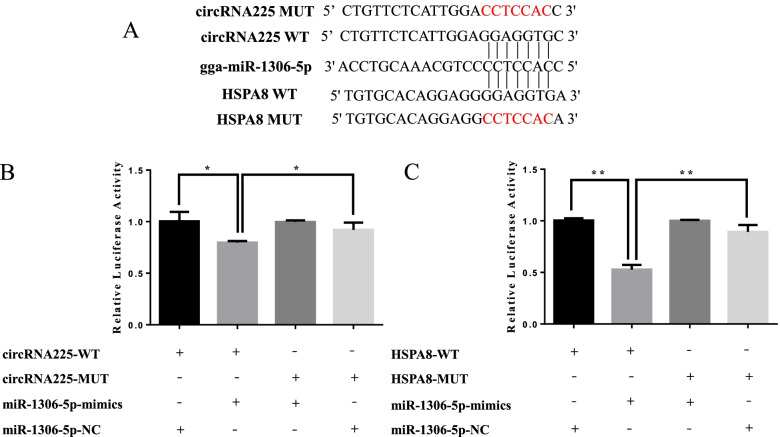
Experimental verification of the circRNA225-gga-miR-1306-5p-HSPA8 regulatory interaction. **A** Schema of miR-1306-5p binding site in chicken circRNA225 and HSPA8 3’UTR sequence. **B** Luminescence was measured after co-transfecting the wild-type or mutant sequence of circRNA225 with miR-1306-5p mimics (or mimics negative control (NC)) in 293 T cells. **C** Luminescence was measured after co-transfecting the wild-type or mutant sequence of HSPA8 3’UTR with miR-1306-5p mimics (or mimics NC) in 293 T cells

Our network analysis predicted that there might be ceRNA regulation among circRNA225, gga-miR-1306-5p, and HSPA8 (Fig. 6A). The expression of circRNA225 and HSPA8 were upregulated with age (Table S3, Table S4), while the expression of gga-miR-1306-5p was downregulated with age (Table S8). Therefore, the target relationship of these transcripts was validated using a luciferase reporter gene assay. As demonstrated in Fig. 6B, gga-miR-1306-5p significantly reduced the firefly luciferase activity of the wild type of the circRNA225 reporter compared with negative control, suggesting that circRNA225 directly targets chicken gga-miR-1306-5p. Meanwhile, the target relationship between gga-miR-1306-5p and HSPA8 also showed that gga-miR-1306-5p could combined with the site of wild type reporter, but not the mutant type reporter (Fig. 6C).

## Discussion

Studying the mechanism underlying skeletal muscle development would be beneficial to the genetic improvement of the quality and quantity of meat. Therefore, the most critical goal of meat production science is analyzing and understanding the development of muscles. Precisely timed gene expression and alternative splicing patterns play important roles in muscle development. Back-splicing events lead to a large number of specific circRNA species, some of which have important regulatory potential in muscle development [29, 30]. In chicks, the circRNA expression profile of embryonic leg muscle (E 11, E 16, and D 1) was uncovered, and 13,377 circRNAs were identified with 462 DE-circRNAs [9]. In the present study, we explored the circRNA expression profile of chicken breast muscle from embryonic (E 12, E 17, and D 1) to post-hatching (D 14, D 56, and D 98) stages and identified 5,755 DE-circRNAs (*p*-value < 0.05, log_2_(fold change) ≥ 1 or log_2_(fold change) ≤ -1). Our study identified many circRNAs that were highly expressed after birth and found many differences in circRNA expression at different stages before and after birth. For example, there are fewer DE-circRNAs within hatching periods (e.g., E 17 versus E 12 or D 1 versus E 17) or post-hatching periods (e.g., D 98 versus D 56 or D 56 versus D 14), but abundant DE-circRNAs between the hatching period and the post-hatching period (e.g., D 98 versus E 12 or D 14 versus E 17). Moreover, muscle tissue was also increased rapidly after hatching. Therefore, our results may provide a novel explanation for the phenotype of the differences in muscle development between the embryonic period and the post-hatching period at circRNA expression levels.

**Fig. 7:**
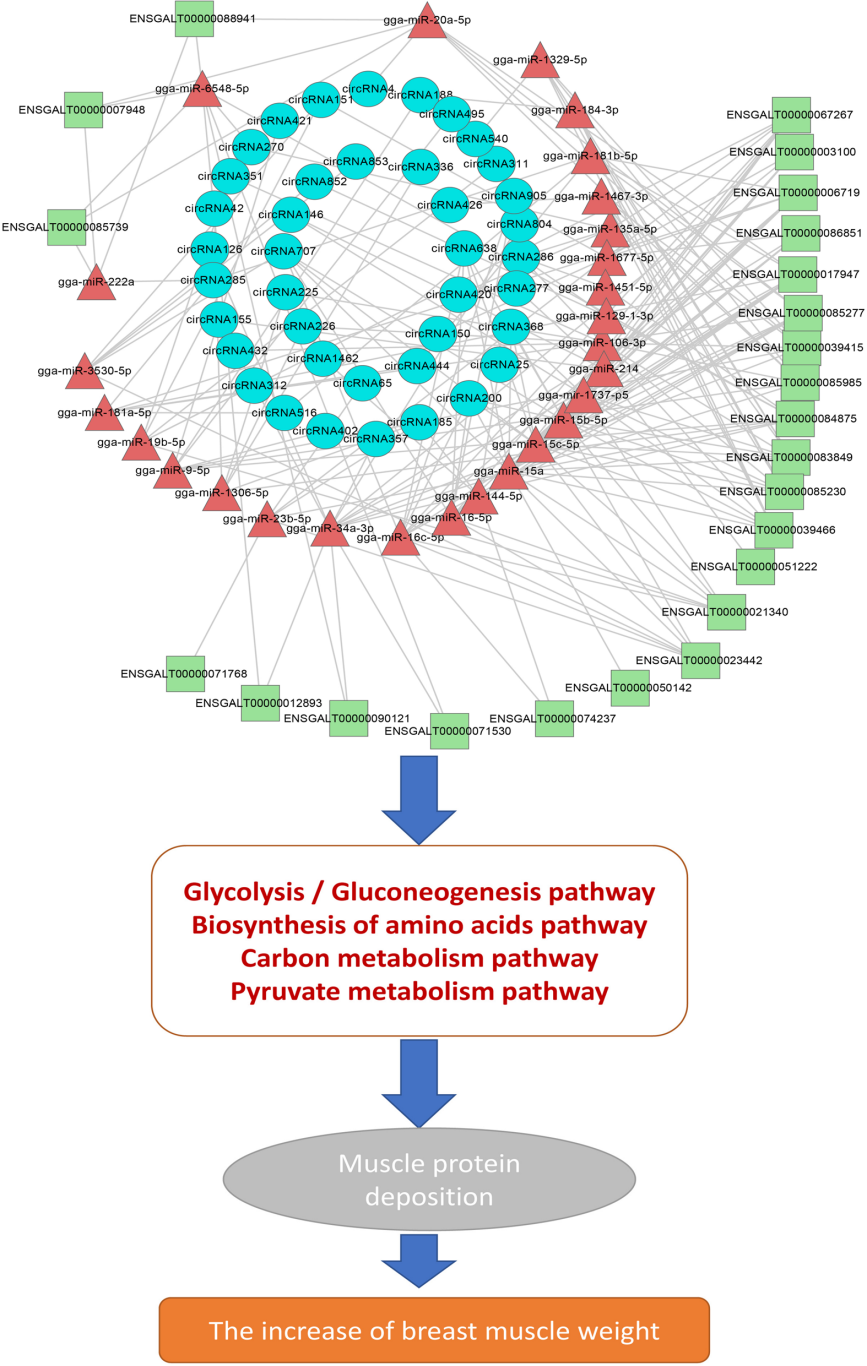
The new circRNA-miRNA-mRNA regulatory network for muscle development of chichken. Blue circles represent circRNAs, red triangles represent miRNAs, and green rectangles represent mRNAs

The main mechanism of circRNA may be in acting as miRNA sponges to modulate post-transcriptional regulation [31]. Our previous studies have explored mRNA and miRNA expression at the same time points as the present research [11, 28]. Therefore, we performed circRNA-miRNA-mRNA regulatory network construction using the circRNAs and mRNAs with the same expression pattern and the 587 miRNAs identified in the chicken muscle in our previous studies. As a result, we obtained a circRNA-miRNA-mRNA regulatory network consisting of 68 circRNAs, 599 mRNAs, and 361 miRNAs, and these circRNAs and mRNAs showed a tendency of upregulation with an increase in age. Functional analysis indicated that these up-regulating circRNAs took part in muscle development through regulating genes in muscle metabolism, such as glycolytic processes, gluconeogenesis, AMP-activated protein kinase activity, glycolysis/gluconeogenesis, biosynthesis of amino acids, and pyruvate metabolism. A previous study also showed that genes associated with the biosynthesis of amino acids, glycolysis/gluconeogenesis, and the tricarboxylic acid (TCA) cycle were upregulated during the goat muscle development [32]. Studies in poultry have shown that muscle mass increased by hypertrophy (increased cellular protein content) after hatching, and this process was controlled by the muscle protein synthesis and degradation [33]. Protein metabolism is often accompanied by energy metabolism, and the glycolysis/gluconeogenesis produces the energy needed for protein turnover during skeletal muscle development [34]. Meanwhile, carbohydrate metabolism can provide a carbon skeleton for nonessential amino acid synthesis. Our ceRNA network analysis also showed that several circRNAs not only play important roles in glycolysis/gluconeogenesis but also contribute to amino acid synthesis through the ceRNA regulation after hatching, which is consistent with the phenotype of rapid increase of muscle tissue and muscle fiber diameter after hatching. Therefore, our study once again demonstrated that the rapid increase in muscle tissue after hatching may be due to the activation of pathways associated with muscle protein deposition, suggesting that ensuring proper energy and protein nutrition after hatching is critical for chicken production (Fig. 7).

Interestingly, we observed that two circRNA isoforms (circRNA225 and circRNA226) from the GPD2 gene in this regulatory network. circRNA225 and circRNA226 co-regulated seven miRNAs (gga-miR-106-3p, gga-miR-183, gga-miR-12239-5p, gga-miR-12283-3p, gga-miR-1306-5p, gga-miR-1773-3p, and gga-miR-3594-3p) and 207 mRNAs (e.g., *PKM*, *PGAM1*, *TPI1*, *LDHA*, *UCP3*, *ATP1A1*, *HSPA8*, and *CHAC1*) through ceRNA regulation (Fig. S1). The miR-183 family gene cluster plays multiple roles in a wide range of physiological and pathological processes, such as cell proliferation, apoptosis, and metabolism [35]. The overexpression of miR-183-5p inhibits the myogenic differentiation of C2C12 myoblasts [36]. MiR-106b has been found to regulate cellular cholesterol efflux by targeting ABCA1 in macrophages [37], miR-106 plays a role in various dystrophies such as facioscapulohumeral muscular dystrophy, limb-girdle muscular dystrophies, and Miyoshi myopathy [38]. Genes, such as Pyruvate kinase muscle (PKM), Phosphoglycerate mutase 1 (PGAM1), triosephosphate isomerase (TPI1), or lactate dehydrogenase A (LDHA), encoding key enzymes involved in gluconeogenesis/glycolysis and biosynthesis of amino acids KEGG pathway, were regulated by circRNA225 and circRNA226. Pyruvate kinase muscle (PKM) is a key enzyme propelling glycolysis in muscle [39]. Proliferating C2C12 cells exhibit preferential transcription of the PKM2 splice isoform, which is proposed to be essential for the generation of sufficient intermediates in cells for the generation of new macromolecules [40]. Phosphoglycerate mutase 1 (PGAM1) reversibly catalyzes a unique step during glycolysis, controlling the metabolite levels of its substrate in the later stages of the glycolytic pathway [41]. The expression levels of the PGAM1 protein in muscle was positively correlated with chicken aging [42]. Comparison of the longissimus thoracis muscle transcriptome from 15- and 19-month-old Charolais bull calves selected divergently for high or low muscle growth revealed that about two-thirds of the genes (including TPI1 and LDHA) involved in glycolysis were upregulated at 15 and 19 months of age in high muscle growth bull calves. Moreover, other genes that were regulated by circRNA225 and circRNA226 were also involved in muscle metabolism and affected muscle development. Uncoupling proteins (UCPs) can uncouple ATP production from mitochondrial respiration, thereby dissipating energy as heat and affecting energy metabolism efficiency [43]. UCP3, a fatty acid anion exporter, supports high rates of fatty acid oxidation in muscles [44]. The Na/K-ATPase (NKA) α1 isoform is encoded by the ATPase Na + /K + transporting subunit alpha 1 (ATP1A1) gene. A skeletal muscle-specific ablation of NKA α1 mice had a 35% reduction in skeletal muscle mass and a switch from oxidative to glycolytic fibers [45]. Cation transport regulator-like protein 1 (CHAC1) is a pro-apoptotic protein that has γ-glutamylcyclotransferase activity. The complete knockout of CHAC1 is embryonic lethal. CHAC1-heterozygote (het) mice have decreased muscle mass [46]. Heat shock protein alpha 8 (HSPA8) is a molecular chaperone and a member of the heat shock protein family that plays an integral role in amino compound metabolism and lipid homeostasis [47]. The expression levels of HSPA8 in 6 weeks old Pekin Duck were significantly higher than at 2- and 4-weeks-old [48], which was similar with our result. Our experiment results showed that there were target sites of gga-miR-1306-5p in the circRNA225 sequence and HSPA8 mRNA 3´-UTR. Therefore, circRNA225 and circRNA226 may be the potential key factors in the regulation of muscle development by regulation of these miRNAs and mRNAs involved in metabolism.

## Conclusion

In summary, our study revealed the expression profiles and potential functions of circRNAs in the breast muscle development of chicken. In total, 5,755 DE-circRNAs were identified during muscle development. We profiled the expression of DE-circRNAs and DE-mRNAs (identified in our previous study) at up to six time points during chicken muscle development and uncovered a significant profile (profile 16) for circRNA upregulation during aging in muscle tissues. We then constructed a circRNA-miRNA-mRNA regulatory network using the circRNAs and mRNAs in profile 16 and miRNAs identified in our previous study. These circRNAs mainly contribute to metabolism in muscle development by ceRNA regulation. Our study suggested that postnatal nutrient regulation is critical for chicken production and identified many circRNAs that influence muscle development by regulating muscle metabolism.

## Supplementary Information

**Additional file 1:** **Table S1.** Detailed information of primers used in this study.

**Additional file 2:** **Table S2.** The expression of all identified circRNAs. 

**Additional file 3:** **Table S3.** The differentially expressed circRNAs.

**Additional file 4:** **Table S4.** The DE-mRNAs identified in our previous study.

**Additional file 5:** **Table S5.** The circRNAs and mRNAs identified in profile 3.

**Additional file 6:** **Table S6.** The circRNAs and mRNAs identified in profile 16.

**Additional file 7:** Table S7. The circRNAs and mRNAs identified in profile 14.

**Additional file 8:** **Table S8.** The miRNAs identified in our previous study.

**Additional file 9:** **Table S9.** The miRNA-circRNA interaction pairs and miRNA-mRNA interaction pairs in profile 16.

**Additional file 10:** **Table S10.** The circRNA-miRNA-mRNA network of profile 16.

**Additional file 11:** **Table S11. **The circRNA-miRNA-mRNA network for circRNA225 and circRNA226.

**Additional file 12:** **Table S12.** Function annotation of the circRNAs in profile 16.

**Additional file 13:** **Figure S1.** Predicted biomathematical circRNA-miRNA-mRNA network for circRNA225 and circRNA226. Only the top 50 mRNAs are shown in the network. Yellow ellipses represent circRNAs, green diamonds represent miRNAs, and red triangles represent mRNAs.

## Data Availability

The raw sequence data reported in this paper have been deposited in the Genome Sequence Archive in BIG Data Center, Beijing Institute of Genomics (BIG), Chinese Academy of Sciences and is publicly accessible at http://bigd.big.ac.cn/gsa (accession no CRA002573 (circRNA), CRA002587 (miRNA), CRA001773 (mRNA)).

## Abbreviations

ATP1A1: ATPase Na + /K + transporting subunit alpha 1
CHAC1: Cation transport regulator-like protein 1
GPD2: Glycerol-3-phosphate dehydrogenase 2
HSPA8: Heat shock protein alpha 8
LDHA: Lactate dehydrogenase A
PGAM1: Phosphoglycerate mutase 1
PKM: Pyruvate kinase muscle
TPI1: Triosephosphate isomerase
UCP3: Uncoupling protein 3
