# Supplementary material for: Integrative analysis of circRNA, miRNA, and mRNA profiles to reveal ceRNA regulation in chicken muscle development from the embryonic to post-hatching periods
Source: BMC Genomics. 2022 May 3;23:342. doi: 10.1186/s12864-022-08525-5 (PMC9063329; doi:10.1186/s12864-022-08525-5)
Supplement: Supplementary file 13 — Additional file 13: Figure S1. Predicted biomathematical circRNA-miRNA-mRNA network for circRNA225 and circRNA226. Only the top 50 mRNAs are shown in the network. Yellow ellipses represent circRNAs, green diamonds represent miRNAs, and red triangles represent mRNAs. [file 12864_2022_8525_MOESM13_ESM.pdf]

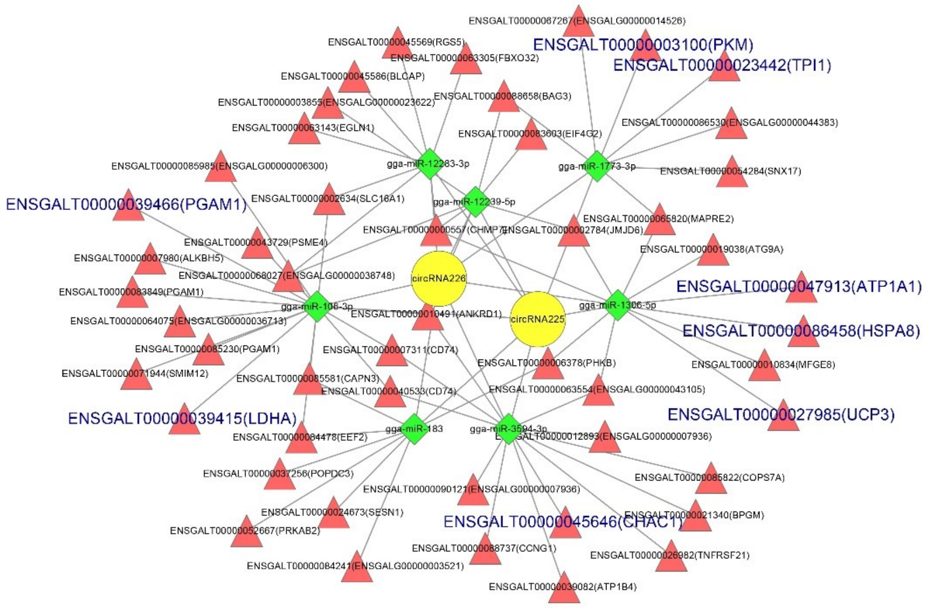

Figure S1. Predicted biomathematical circRNA-miRNA-mRNA network for circRNA225 and circRNA226. Only the top 50 mRNAs are shown in the network. Yellow ellipses represent circRNAs, green diamonds represent miRNAs, and red triangles represent mRNAs.
